# Antidepressant Use for Improving Functional Ischemic Stroke Outcomes

**DOI:** 10.7759/cureus.5908

**Published:** 2019-10-14

**Authors:** Caleb J Heiberger, Clayton Busch, Kevin Rance, Brett Montieth, John Chandler, Josh Hanscom, Stephanie Kazi, Divyajot Sandhu, Gauravjot Sandhu, Tej I Mehta

**Affiliations:** 1 Radiology, University of South Dakota Sanford School of Medicine, Sioux Falls, USA; 2 Neurology, University of South Dakota Sanford School of Medicine, Sioux Falls, USA; 3 Anesthesiology, University of South Dakota Sanford School of Medicine, Sioux Falls, USA; 4 Family Medicine, University of South Dakota Sanford School of Medicine, Sioux Falls, USA; 5 Emergency Medicine, University of South Dakota Sanford School of Medicine, Sioux Falls, USA; 6 Internal Medicine, University of South Dakota Sanford School of Medicine, Sioux Falls, USA; 7 Neurology, Sanford Health, Sioux Falls, USA

**Keywords:** ischemic stroke, antidepressant, stroke, functional

## Abstract

Objective

To assess the effect of antidepressants on functional post-stroke recovery, we conducted a retrospective analysis among acute ischemic stroke patients with a subgroup analysis of severe stroke cases, assessing outcomes through 18 months.

Methods

A retrospectively gathered ischemic stroke population was obtained from an institutional database. Grouping was via intention-to-treat with antidepressant use post-stroke or lack thereof. Patients with severe stroke (NIHSS ≥ 21) were further analyzed independently. The primary and secondary outcomes were modified Rankin scale (mRS) and survival over 18 months, respectively. Patient demographics and NIHSS were obtained. Data were analyzed in R using adjusted logarithmic-multivariate models. Adjusted Cox proportional hazards models were used to estimate associations between survival and antidepressants.

Results

Eight-hundred six patients (52 severe strokes) received antidepressants post-stroke while 948 (56 severe) did not. The antidepressant group was more female (56% to 43.5%) and had significantly better survival rates (88% vs. 79%, HR 0.62, p < 0.01) but not mRS scores (2.13 vs 2.24, p = 0.262) by the end of the study period. Among severe stroke cases, those receiving antidepressants showed better survival rates (79% vs. 60%, HR 0.36, p=0.026) and most recent mRS score (3.9 vs 5, p < 0.01). The analysis controlling for demographics variables retained significance.

Conclusion

Antidepressant use post-stroke may improve functional outcomes in patients suffering from severe stroke and may decrease all-cause mortality for strokes of any severity.

## Introduction

Ischemic stroke is a significant cause of morbidity and mortality worldwide [1]. Various antidepressant medications, namely, selective serotonin reuptake inhibitors (SSRIs), have demonstrated efficacy at enhancing post-stroke recovery. In 2011, the “FLuoxetine for motor recovery After acute ischaeMic strokE” (FLAME) trial reported enhanced motor recovery post-stroke among patients given 20 mg fluoxetine daily versus placebo over three months [2]. Subsequent meta-analyses of additional placebo-controlled trials post-stroke found similar results to the FLAME trial, correlating fluoxetine treatment with greater independent living functions (mRS of 0-2). These collective results were called into question with the recent “Effects of fluoxetine on functional outcomes after acute stroke” (FOCUS) trial, which did not find significant improvements in functional outcomes among patients given 20 mg fluoxetine daily over six months of follow-up [3]. The trial did, however, find a decreased incidence of depression among patients receiving fluoxetine and increased incidence of bone fractures [3].

In 2015, adopting a policy influenced by the FLAME trial, our institution incorporated SSRIs into the standardized care of ischemic stroke patients. Given the contradictory outcomes of the FOCUS and FLAME trials, we conducted a cohort study on ischemic stroke patient populations before and after the standardized use of SSRIs at our institution to better elucidate the potential positive outcomes attributed to antidepressants use post-stroke.

## Materials and methods

Study design

A pre-existing institutional database of patients treated for stroke at a tertiary care center was acquired by querying the electronic medical record for inclusion criteria of ischemic stroke in the past four years (October 1, 2013, and May 27, 2017). The total service population is composed of over 500,000 patients at a major urban healthcare center.

Patients were divided into two groups based on the institutional electronic medication administration record showing a verified prescription and receipt of antidepressant therapy post-stroke or the lack thereof. The “treatment” group was identified as those receiving antidepressants post-stroke and the “control” group was identified as those not receiving antidepressants post-stroke verified by pharmacy information. Due to the high frequency of concomitant SSRI use with other antidepressants, the incidence of all antidepressant uses following ischemic stroke was pooled as one. Patients with a standing prescription for antidepressants post-stroke were included in the treatment group. Among the treatment group, all patients were on antidepressants within one-week post-stroke. A subgroup analysis for patients suffering from severe stroke (defined as NIHSS ≥ 21) was also conducted.

Prescriptions were queried from the medication administration record associated with each patient’s unique electronic medical record (EMR) identifier. All queried data in the study were screened for duplicates, missingness, inaccurate/impossible dates, and incorrect diagnoses. Inconsistencies in reported data were verified via manual chart reviews by two independent researchers. Instances of disputes were settled by a third researcher.

Institutional review board approval was requested and obtained for this study.

Inclusion and exclusion criteria

Cases of reported ischemic stroke with associated NIHSS and mRS scores were included in the study. Any cases other than ischemic stroke, those without NIHSS or mRS data, and vulnerable groups, including pregnant women and those under the age of 18, were excluded.

Group demographics

Demographic data, including age, race, follow-up time, survival status, and gender, were recorded. The first NIHSS (at initial stroke presentation), last NIHSS prior to discharge, first modified Rankin Scale (mRS) score (at time of presentation), and last mRS score (at last follow-up) were acquired. Psychiatric comorbidities (substance abuse, psychotic disorder, personality disorder, nicotine dependence, mood disorder, dementia, bipolar disorder, anxiety, alcohol abuse, and “other” psychiatric disorders) were also recorded. All preceding values and diagnosis (using International Statistical Classification of Diseases and Related Health Problems (ICD)-10 codes) were queried from the EMR linked to unique patient identifiers.

Outcome comparisons

The primary outcome measures were last recorded mRS score and change in mRS score between the first and last recorded values. The secondary outcome measures were survival to the end of the study period (18-month follow-up) and incidence of depression following stroke. Charts identified to have a diagnosis of depression when queried using associated ICD-10 codes (F33) were subsequently manually reviewed independently by two researchers to identify active depressive episodes within 18 months from the time of stroke, as documented by a providing physician. Disputes were settled by the involvement of a third researcher.

Statistical analysis

Statistical analysis was performed using R. For univariate analyses, differences in continuous mean scores of demographic data and explanatory measures between the dichotomous summary measure of antidepressant use versus no antidepressant use were examined using independent t-tests for normally distributed data and the Mann-Whitney U test for skewed data. A multivariate model adjusted for confounding variables was generated to determine the significance and effect size of antidepressant use on mRS scores. Kaplan-Meir survival curves were generated to examine survival differences among patients taking and not taking antidepressants. Patients were censored if study participation was terminated for reasons other than death. Cox proportional hazard modeling was conducted to determine hazard ratios (HR) and adjusted hazard ratios (aHR) for survival analysis.

## Results

Study cohort demographics

Two-thousand twenty-two patients were identified as suffering from acute ischemic stroke, 1754 of which had data regarding the primary and secondary outcomes. Age ranged between 21 and 101, with a mean of 69.7. The follow-up time mean was 409.4 days, with a range of 0 to 548 days. Forty-nine percent of patients were female and 51% were male. One-thousand one-hundred seventy-five patients had data available regarding race/ethnicity, the preponderance of whom was Caucasian (1106). The mean first recorded and last recorded NIHSS were 5.4 and 5.33, respectively. Among all stroke survivors, there was a significantly increased incidence of recorded neuropsychiatric histories in the antidepressant group with the exceptions of alcohol abuse, nicotine dependence, and bipolar disorder (although bipolar disorder was exactly at the level of significance). Demographics for the study cohort are displayed in Table 1.

**Table 1:** Characteristics and outcomes of the study cohort NIHSS: National Institute of Health Stroke Scale: mRS: modified Rankin scale

Characteristic	No antidepressant	Antidepressant	p-value
n	948	806	-
Mean age (sd)	69.92 (15.8)	69.44 (11.2)	0.525
Male (%)	536 (56.5)	355 (44.0)	< 0.001
Mean first recorded NIHSS (sd)	5.74 (7.5)	5.14 (6.8)	0.094
Mean last recorded NIHSS (sd)	5.61 (7.5)	5.06 (6.7)	0.129
Mean first recorded mRS (sd)	2.41 (1.7)	2.37 (1.8)	0.677
Mean last recorded mRS (sd)	2.24 (2.0)	2.13 (1.8)	0.262
Mean change in mRS (sd)	0.17 (1.3)	0.24 (1.3)	0.253
Depression post-stroke (%)	81 (8.5)	30 (3.7)	<0.001
Alcohol abuse (%)	90 (9.5)	69 (8.6)	0.5
Anxiety disorder (%)	199 (21.0)	429 (53.2)	<0.001
Bipolar disorder (%)	6 (0.6)	13 (1.6)	0.05
Dementia (%)	106 (11.2)	139 (17.2)	<0.001
Nicotine dependence (%)	294 (31.0)	282 (35.0)	0.08
Other psychiatric disorder (%)	31 (3.3)	58 (7.2)	<0.001
Psychotic disorder (%)	65 (6.9)	93 (11.5)	0.006
Personality disorder (%)	11 (1.2)	22 (2.7)	0.015
Substance abuse (%)	48 (5.1)	67 (8.3)	0.006

The first-recorded mRS and last-recorded mRS means were 2.4 and 2.2, respectively. Average antidepressant dose for antidepressant subclasses and percent of antidepressant cohort receiving each class of medication are presented in Table 2.

**Table 2:** Antidepressant dosage and percent of sample covered SSRI: selective serotonin reuptake inhibitors; SNRI: serotonin and norepinephrine reuptake inhibitors

Drug Class	Average Dosage	Percentage Using
Alpha-2 Receptor Antagonists	18 mg	11.0
Antidepressant – Misc.	168 mg	8.6
SSRI	32 mg	70.7
Serotonin Modulators	65 mg	12.7
SNRI	69 mg	12.5
Tri-Cyclic	37 mg	10.9

Outcome comparisons study cohort

Of this original group, 806 were identified as using antidepressants post-stroke (the treatment group) and 948 were identified as not using antidepressants post-stroke (the control group). There were no significant differences in the primary outcome measures between the treatment and control groups (last recorded mRS score 2.13 and 2.24, respectively, p = 0.262), nor was there a significant difference in the change in mRS score between the two groups (-0.24 and -0.17, respectively, p = 0.262).

Multivariate analysis controlling for age, sex, and the development of depression post-stroke did not find a significant effect of antidepressant use in the post-stroke time period on mRS score (adjusted mRS score among those not receiving antidepressants of 2.05 and among those receiving antidepressants of 1.82, p = 0.21). Multivariate analysis controlling for the same factors likewise did not find a significant effect of antidepressant use in the post-stroke time period on the change in mRS score between the two groups.

**Figure 1:**
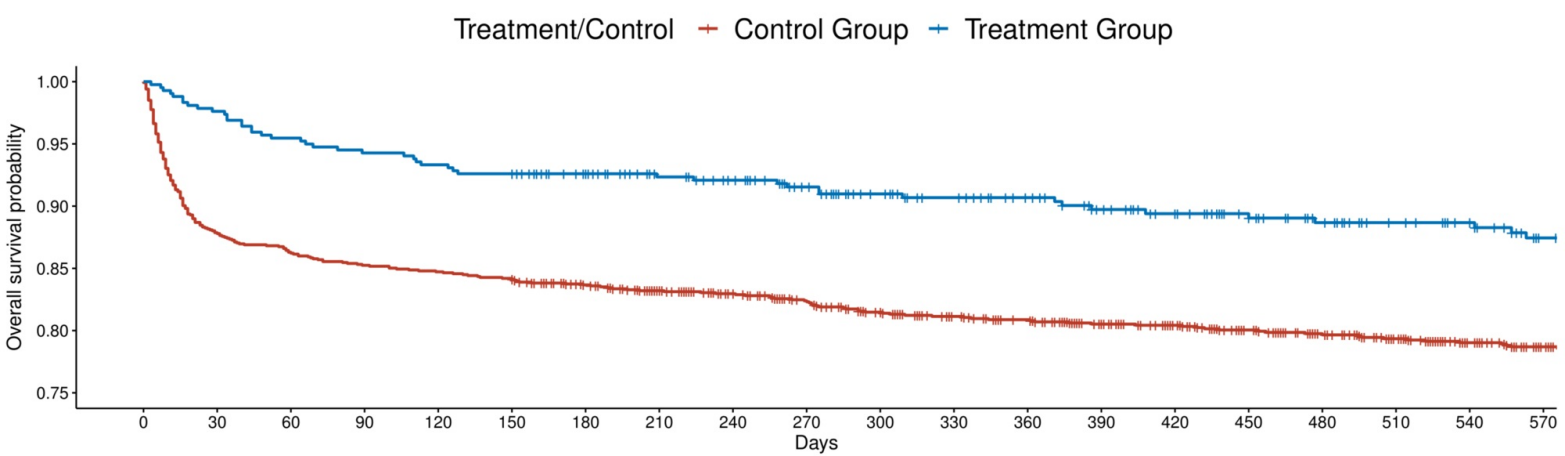
Survival plot of all stroke patients by antidepressant use post-stroke

For the secondary outcome measure, there was a significant difference in survival at 18 months of follow-up, with 79% of the control group and 88% of the treatment group surviving (HR 0.62, 95% CI (0.47 - 0.81), P<0.01 and aHR (controlling for age, sex, and the development of depression post-stroke) 0.64, 95% CI (0.49 - 0.85), P<0.01) (Figure 1). 3.7% of those receiving antidepressants developed depression post-stroke and 8.5% of those not receiving antidepressants developed depression post-stroke (p<0.01). Outcomes for the study cohort are displayed in Table 1.

Severe stroke cohort demographics

Ninety-eight stroke survivors with a first recorded NIHSS greater than or equal to 21 were identified. The mean patient age in this group was 74.84 with a range of 31 to 99. The mean follow-up time was 312.52 days. Fifty-seven percent was female and 43% was male. The mean first and last recorded NIHSS were 25.92 and 25.19, respectively. The mean first-recorded mRS and last-recorded mRS were 4.8 and 4.5, respectively. There were no significant differences in neuropsychiatric histories within this group. Demographics for severe strokes are displayed in Table 3.

**Table 3:** Characteristics and outcomes of severe stroke cohort NIHSS: National Institute of Health Stroke Scale; mRS: modified Rankin scale

Characteristic	No antidepressant	Antidepressant	p-value
N	56	42	-
Mean age (sd)	77.98 (15.0)	70.64 (13.0)	0.013
Male (%)	24 (44.6)	17 (40.5)	0.68
Mean first recorded NIHSS (sd)	26.43 (4.1)	25.24 (3.8)	0.149
Mean last recorded NIHSS (sd)	26.16 (4.5)	23.90 (5.9)	0.034
Mean first recorded mRS (sd)	4.80 (0.4)	4.79 (0.5)	0.84
Mean last recorded mRS (sd)	5.00 (1.3)	3.93 (1.8)	0.001
Mean change in mRS (sd)	0.20 (1.4)	-0.86 (1.8)	0.002
Post-Stroke Depression (%)	2 (3.6)	1 (2.4)	0.74
Alcohol Abuse (%)	5 (8.9)	6 (14.3)	0.41
Anxiety Disorder (%)	8 (14.3)	11 (26.2)	0.14
Bipolar Disorder (%)	1 (1.8)	0 (0.0)	1
Dementia (%)	6 (10.7)	5 (11.9)	0.85
Nicotine Dependence (%)	11 (19.6)	15 (35.7)	0.18
Other psychiatric Disorder (%)	0 (0.0)	3 (7.1)	0.08
Psychotic Disorder (%)	2 (3.6)	3 (7.1)	0.43
Personality Disorder (%)	0 (0.0)	2 (4.8)	0.43
Substance Abuse (%)	2 (3.6)	2 (4.8)	0.77

Outcome comparisons for severe strokes

Among stroke survivors with NIHSS ≥ 21, 42 were identified as using antidepressants post-stroke (treatment group) and 56 were not (control group). There were significant differences in the primary outcome measures between last recorded mRS score of the treatment (3.9) and control group (5.0, p<0.01). The mean change in mRS score was -0.86 for the treatment group and 0.20 for the control group (p<0.01). There was also a significant difference in the last recorded NIHSS of the treatment (23.9) and control groups (26.16).

Multivariate analysis controlling for age, sex, last recorded NIHSS, and the development of depression post-stroke additionally found a significant, positive effect of antidepressant use in the post-stroke time period on mRS score (adjusted mRS score among those not receiving antidepressants of 2.67 and among those receiving antidepressants of 2.19, p<0.01). Multivariate analysis controlling for the same factors showed evidence for a significant advantageous effect of antidepressant use on change in mRS scores with antidepressant use decreasing mRS scores by -0.81 units over 18 months of follow-up compared to no antidepressant use (p<0.01).

**Figure 2:**
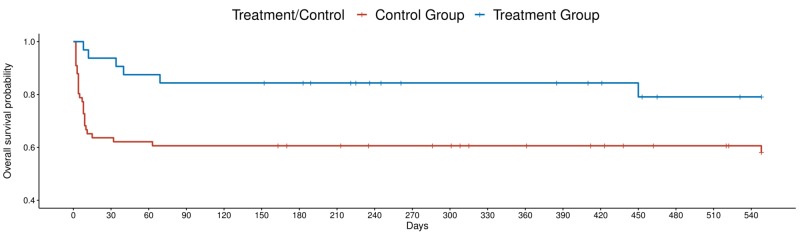
Survival plot of severe stroke patients by antidepressant use post-stroke

A significant difference in survival rates between those receiving (79%) and those not receiving antidepressants (60%) was evident through 18 months (HR 0.36, p=0.026 and aHR 0.39, p=0.04, controlling for age, sex, and the development of depression post-stroke) (Figure 2). There was no significant difference in the incidence of depression among severe post-stroke survivor groups. Outcomes for severe strokes are displayed in Table 3.

## Discussion

The results of this study indicate that antidepressant use post-stroke is associated with increased survival over 18 months of follow-up among all ischemic stroke patients, including patients with severe stroke (Figure 1 and Figure 2). Further, there were significantly lower mRS scores among patients with severe stroke receiving antidepressants over 18 months of follow-up. However, there was no significant difference in mRS between those receiving and not receiving antidepressants among all stroke patients.

These data seem to strike a balance between the FOCUS and FLAME trials. While there were no significant changes in mRS among all stroke patients, as in the FOCUS trial, there were significant changes in mRS among patients with NIHSS ≥ 21. Moreover, increased survival among patients taking antidepressants over 18 months was evident in both groups. Finally, the incidence of depression among all patients receiving antidepressants in our study was less than that of patients not receiving antidepressants, akin to the FOCUS trial, but this result did not hold for patients with severe strokes.

Stroke survivor demographics for the entire stroke population in our study were in keeping with the underlying population, however, there was a significant correlation between sex and antidepressant use post-stroke, with women significantly more likely to use antidepressants. Bias on the prescription of antidepressants per gender post-stroke may be related to previous studies indicate women are more likely to suffer from post-stroke depression [4]. Generally, there were significant correlations between antidepressant use post-stroke and a past medical history of neuropsychiatric disorders [4-7]. In previous research, increased psychological distress has been associated with heightened stroke risk. Further, premorbid psychiatric conditions predict post-stroke psychiatric conditions that are associated with worse functional and cognitive outcomes [5,7]. Therefore, the premorbid psychiatric history of patients may have influenced post-stroke antidepressant treatment. Despite associated worsened outcomes with psychopathologies, patients in the antidepressant group had better functional outcomes.

Additionally, there was a significant difference in age between the treatment and control groups among severe stroke survivors with the control group being approximately an average of seven years older than the treatment group. This difference could confound both the likelihood of antidepressant prescription, the difference in mRS, and the difference in survival between the two groups. While literature supports the deleterious influence of age on post-stroke cognitive outcomes, the impact on functional outcomes has been reported as small enough in size to be of questionable clinical significance [8-9]. Further, multivariant analysis controlling for age, sex, last recorded NIHSS, and the development of depression indicated an advantageous effect from antidepressants on mRS outcomes and survival in severe strokes despite the age difference between cohorts.

This study is not without limitations. Owing to its retrospective design and lack of randomization, bias on behalf of the researchers cannot be excluded. Additionally, while this study was of the intention-to-treat design and we collected pharmacy data from within our institution, extraneous prescriptions from outside institutions could not be adequately assessed. To go along with this, and the frequent concomitant use of SSRIs with other antidepressants, we elected to pool and analyze all antidepressant recipients together in order to increase the statistical power of this study (Table 3). However, this precludes us from making specific recommendations regarding the choice of antidepressant and dosage. The methodological inferiority of this study compared to the preceding FLAME and FOCUS trials limit its clinical decision guiding capacity. However, considering the nearly equivalent methodological soundness and conflicting results of the FLAME and FOCUS trials further investigation is warranted, to which this study may serve as an initial step.

Future research focusing on the impact of specific antidepressant drug classes and dosages on various stroke populations would provide valuable data for clinical practice. Identifying specific causes of mortality as well as other comorbid conditions developed in the post-stroke window would provide a better sense of the potential adverse effects in treating stroke patients with antidepressants. Clinical trials on antidepressants other than SSRIs may be warranted to explore avenues of treatment not studied in the FLAME and FOCUS trials.

## Conclusions

Antidepressant use post-stroke may improve functional outcomes in patients suffering severe stroke and decrease all-cause mortality for strokes of any severity, coinciding with a lower incidence of post-stroke depression. Due to the conflicting nature of the FLAME and FOCUS trials, these results serve as an initiative measure to guide ongoing research. Further study is indicated to identify the impact of specific antidepressant classes and dosages on various stroke populations.
